# Research progress and clinical application of all-inside reconstruction techniques: narrative review

**DOI:** 10.3389/fsurg.2025.1644737

**Published:** 2025-09-12

**Authors:** Yong Jiang, Dikun Qian, Qiming Lu, Yangbin Cao, Yi Ren, Jun Su

**Affiliations:** Qiannan Prefecture Traditional Chinese Medicine Hospital, Duyun, China

**Keywords:** all-inside reconstruction, ACL, PCL, clinical application, limitations, perspectives

## Abstract

All-inside reconstruction is a popular technique for cruciate ligament reconstruction in recent years. Compared with traditional reconstruction techniques, all-inside reconstruction is primarily performed through minimally invasive arthroscopic procedures, involving the creation of separate half-tunnels at the femoral and tibial ends, followed by fixation of the graft using adjustable suspension devices to achieve anatomical ligament reconstruction. Current research primarily focuses on anterior cruciate ligament (ACL) reconstruction in the knee joint, demonstrating advantages such as reduced surgical trauma, bone preservation, decreased postoperative pain, and accelerated rehabilitation. Extensive clinical studies have shown that all-inside reconstruction achieves comparable mid- to short-term clinical outcomes to traditional techniques, with superior performance in certain functional metrics. Additionally, all-inside reconstruction is associated with lower complication rates, smaller incisions that better preserve tendons, and reduced postoperative pain and discomfort. However, the technique also has limitations, such as a steep learning curve, potential graft micromotion due to suspension fixation, and tunnel widening. Therefore, this review aims to comprehensively elaborate on the developmental history, principles, applications across various joints, perioperative management, postoperative rehabilitation, efficacy evaluation, and biomechanical research outcomes of all-inside reconstruction. It will also discuss the clinical advantages and limitations based on the latest clinical studies, as well as future directions for improvement and research prospects.

## 1 Introduction

With the rapid advancement of modern arthroscopic techniques and implant materials, there is an increasing demand for improved treatment of ligament injuries. As an innovative technology, all-inside reconstruction has become a major advancement in the field of cruciate ligament reconstruction (1). Traditional ligament reconstruction often requires large bone tunnels and multiple tendon grafts, presenting drawbacks such as high graft demand, significant bone damage, postoperative pain, and slow recovery (2). To address these limitations, all-inside reconstruction was proposed and has progressively matured. Initially achieving breakthroughs in ACL reconstruction of the knee, the technique has since expanded to posterior cruciate ligament (PCL) and ligament/soft tissue reconstruction in other joints (3, 4). Its core feature is the completion of all bone tunnel preparation and graft fixation under arthroscopy, eliminating the need for large percutaneous bone exits, thereby maximizing preservation of bone cortex and soft tissue attachments (5, 6). Due to its minimal invasiveness and high stability, all-inside reconstruction is most commonly used for ACL injuries (7, 8). ACL ruptures affect approximately 175,000 cases annually in the United States, with an incidence rate of about 1/2,000 in Germany (9, 10). As public sports participation increases and imaging diagnostic technologies advance, the incidence of such injuries continues to rise. Improper management of ACL tears may lead to anterior knee instability, secondary meniscal tears, cartilage wear, and even early-onset osteoarthritis. Current clinical approaches primarily include conservative rehabilitation or arthroscopic reconstruction (11). Conservative treatment focuses on restoring function through muscle strengthening, joint mobility, and proprioception training without altering anatomical structures. Anterior cruciate ligament reconstruction (ACLR) utilizes autografts (patellar tendon, quadriceps tendon, or hamstring tendons) to reconstruct the ligament, restoring knee stability, reducing secondary injuries, and delaying cartilage degeneration (12). The recently emphasized all-inside ACLR technique involves drilling closed blind-end bone sockets in the tibia and femur, using tibial suspension fixation, and minimizing skin incisions. A single semitendinosus tendon suffices to achieve a graft diameter >8 mm. In addition, due to individual anatomical differences, some patients have tendons that are inherently thin and limited in length, such that even after being folded four times, the diameter remains less than 8 mm. In such cases, to ensure the strength and stability of the graft, additional tendons must be harvested for supplementation (or the already harvested tendons must be folded six or eight times), and these are either bundled with the original graft or re-woven to achieve the target diameter of ≥8 mm. Studies indicate lower risks of tibial tunnel fracture, better tissue preservation, reduced postoperative pain, faster early functional recovery, and safe application in adolescents with open growth plates, with an overall graft failure rate below 5% (13). However, some longitudinal studies suggest that the incidence of osteoarthritis 10–15 years post-reconstruction may be comparable to or higher than that of conservative treatment, leaving therapeutic strategies still contentious.

This article aims to summarize the current status and clinical applications of all-inside reconstruction technology, including its principles, indications, and contraindications. It will cover practical implementations across different joints, comparisons of surgical techniques, graft selection and fixation methods, radiological and biomechanical evaluations, as well as postoperative rehabilitation and complication management. The advantages and limitations will be analyzed and discussed in light of the latest research findings. All-inside reconstruction is regarded as a revolutionary technique in ligament reconstruction. Particularly in sports medicine, it ensures joint stability while minimizing surgical trauma and accelerating athlete rehabilitation, making it a focal point in clinical treatment. Future advancements in all-inside reconstruction are expected to achieve further breakthroughs in orthopedic ligament injury management.

## 2 Principles and development of all-inside reconstruction techniques

**Figure 1:**
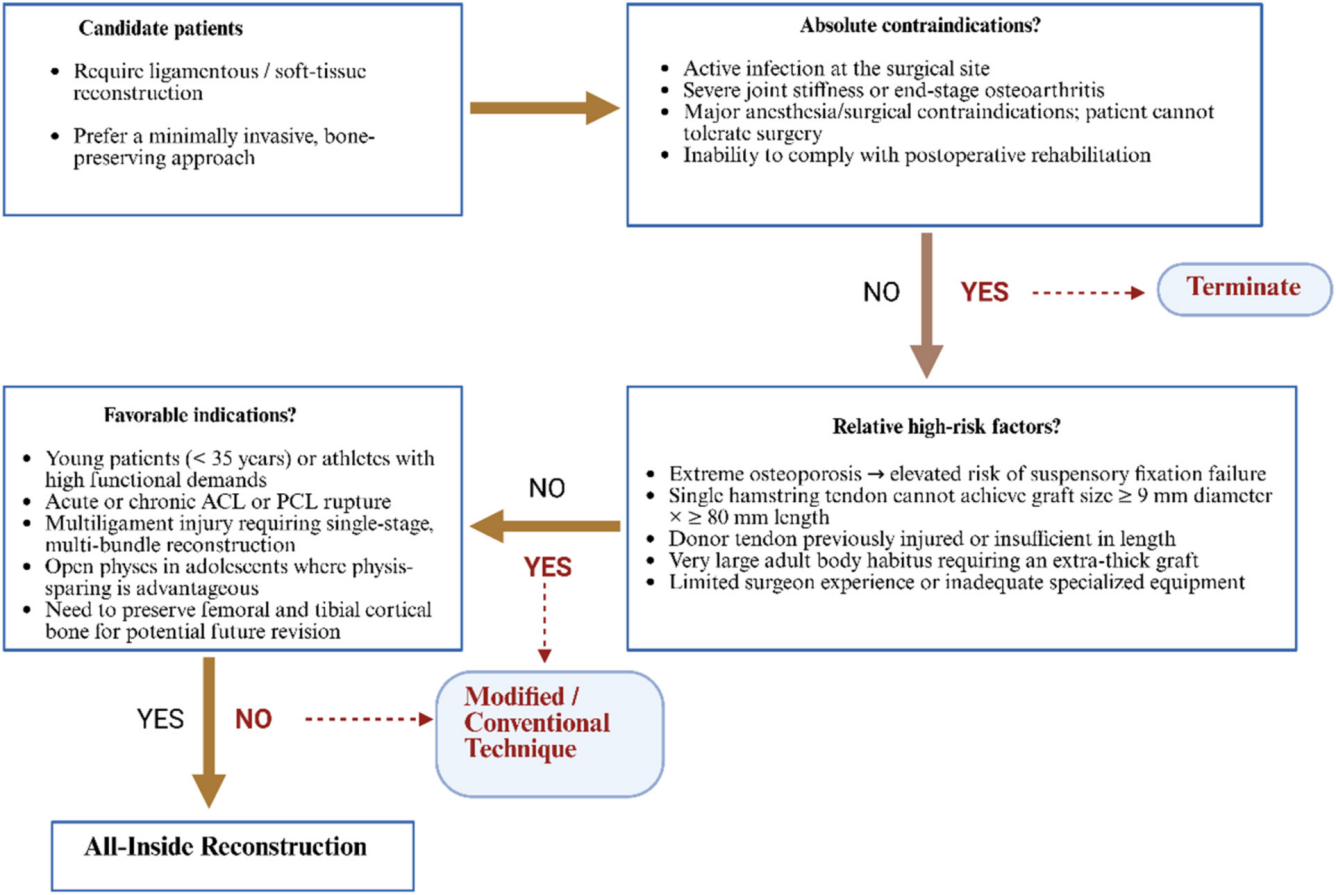
Clinical decision-making algorithm for All-inside reconstruction. The flowchart illustrates a three-step algorithm that guides surgeons in choosing between All-Inside reconstruction and modified/conventional techniques for ligamentous or soft-tissue injuries. (1) Candidate patients: Individuals requiring ligament or soft-tissue reconstruction who prefer a minimally invasive, bone-preserving approach. (2) Absolute contraindications: Active infection at the surgical site, severe joint stiffness or end-stage osteoarthritis, major anesthesia/surgical contraindications, or inability to comply with rehabilitation. Presence of any leads to termination (ellipse). (3) Relative high-risk factors: Extreme osteoporosis; single hamstring tendon cannot achieve a graft ≥9 mm in diameter and ≥80 mm in length; donor tendon previously injured or insufficient; very large body habitus requiring an extra-thick graft; or limited surgical experience/equipment. If yes, select a modified or conventional technique (rectangle). (4) Favorable indications: Young patients (<35 years) or athletes with high functional demands; acute or chronic ACL/PCL rupture; multiligament injury requiring single-stage multi-bundle reconstruction; open physes where physis-sparing is advantageous; or need to preserve femoral/tibial cortex for possible revision. Presence of any directs to All-Inside reconstruction. Diamonds denote decision nodes, rectangles denote treatment choices, and ellipses denote start/termination; solid arrows indicate the main pathway, dashed arrows indicate alternative routes.

All-inside reconstruction refers to the arthroscopic preparation of all bone tunnels and fixation of grafts necessary for ligament reconstruction. The bone tunnels are blindly tunneled at both the femoral and tibial ends, and the ends of the grafts are fixed with suspension devices to the medial cortical bone without penetrating the bone cortex. The key to this technique is the use of special reverse drilling instruments (e.g., flip drill). The medical practitioner arthroscopically flips the drill bit outward from the inside of the bone to precisely prepare the bone groove at the desired depth. Typically, the depth is approximately 15–25 mm at the femoral end and 15–20 mm at the tibial end, which avoids the weakening of the bone structure that would result from a traditional tunnel through the bone (14, 15). At the same time, the graft is usually made of a quadruple folded tendon (autologous semitendinosus tendon is a common choice), which is tied at both ends to a length-adjustable looped button-plate and tightly attached to the inner portion of the bone tunnel, resulting in a strong cortical suspension fixation. Compared with traditional techniques, all-inside reconstruction preserves the lateral cortical integrity of the femur and tibia, resulting in smaller postoperative bone tunnels, a line of force closer to the anatomical position, and a reduced risk of enlarged bone funnels. The concept of “tunnel-less” ACL reconstruction was first proposed by Lubowitz and colleagues (16). Subsequently, in 2011, he and his colleagues further refined the second-generation all-inside ACL reconstruction technique (17). This technique was developed through the introduction of various innovative ideas such as lateral femoral approach slotting, quadruple femoral tendon grafts, rotary drilling, and double-ended adjustable suspension fixation. These improvements have significantly improved the feasibility and stability of the procedure, and have led to the gradual maturation of all-inside reconstruction. Currently, the core principles of All-inside reconstruction have been validated in ACL reconstruction and have been expanded for PCL reconstruction as well as certain joint soft tissue reconstruction (18, 19). In terms of surgical devices and implants, the introduction of the adjustable loop suspension nail is an important support for the total endoprosthesis technique, which can ensure the fixation strength while avoiding interference with the bone cortex and facilitating tension adjustment. Based on minimally invasive instruments and special implants, the anatomical reconstruction and mechanical stabilization of ligaments is achieved by the strategy of “blind tunnel and suspension fixation”, which marks a new stage of arthroscopic reconstruction technology (20, 21). Selection criteria or decision algorithm for all-inside reconstruction in specific patient populations (Figure 1).

## 3 Indications and contraindications for all-inside reconstruction techniques

### 3.1 Indications for all-inside reconstruction technique

The main indication for all-inside reconstruction is for patients with various types of ligamentous injuries who require surgical reconstruction and who wish to minimize trauma. The primary indication is ACL rupture, including acute rupture and old functional instability. All-inside ACL reconstruction is indicated for athletes and younger patients with high demands on knee function, and studies have shown that the all-inside technique achieves good stability and functional recovery in these groups (22, 23). In addition, for multiple ligament injuries (e.g., simultaneous ACL/PCL rupture of the knee), the all-inside technique facilitates the reconstruction of multiple bundles of ligaments in a single operation due to the small bone tunnels and the preservation of the bone volume, avoiding the need for staging or extensive bone grooving in the traditional procedure (24). In PCL reconstruction of the knee, the all-inside technique can solve the challenges such as the difficulty in reaching the tibial stop of traditional PCL reconstruction, and it has become an option for experienced operators (25, 26). The all-inside technique can be considered as an important option in all cases of ligament/soft tissue injuries that are suitable for arthroscopic reconstruction and where surgical trauma reduction is desired.

### 3.2 Contraindications to all-inside reconstruction technique

Contraindications to all-inside reconstruction mainly include the presence of active infection in the surgical area, severe joint stiffness, or advanced osteoarthritis (where the joint has degenerated extensively). It is also contraindicated for patients who cannot tolerate surgery due to medical conditions or who cannot cooperate with postoperative rehabilitation. With respect to the all-inside technique itself, one needs to be alert to relative contraindications (27). Chief among these is the presence of extreme osteoporosis in the patient and the potential increased risk of failure of suspension nail fixation. Second, in the small number of adult giant-sized patients who require extra-thick grafts, the all-inside technique may not be able to meet the graft diameter requirements using only a single tendon. For patients with a history of ipsilateral hamstring donor-site injury or surgery resulting in insufficient semitendinosus tendon length, a single-tendon graft in the standard all-inside technique cannot satisfy the required graft length and diameter. Although one could resort to an allograft or additional tendon harvest, it is more common to preserve the minimally invasive, bone-conserving advantages of the all-inside approach by using combined semitendinosus and gracilis harvest, autologous peroneus longus tendon substitution, a narrow socket all-inside technique with adjustable suspensory fixation, or distal FiberTape augmentation. In addition, in adolescents with unclosed epiphyses, all-inside ACL reconstruction is a relative advantage rather than a contraindication due to the preservation of the epiphysis, reduction of periosteal traversal, and less damage to the epiphysis (28, 29). However, the above maneuvers need to be performed by experienced medical practitioners in order to avoid damage to the epiphyseal plate by drilling too deep into the flipper. Contraindications to all-inside reconstruction are mainly general surgical contraindications and a few special technical limitations, which need to be weighed against the patient's specific situation.

## 4 Clinical application of all-inside reconstruction technique

### 4.1 ACL all-inside reconstruction

**Figure 2:**
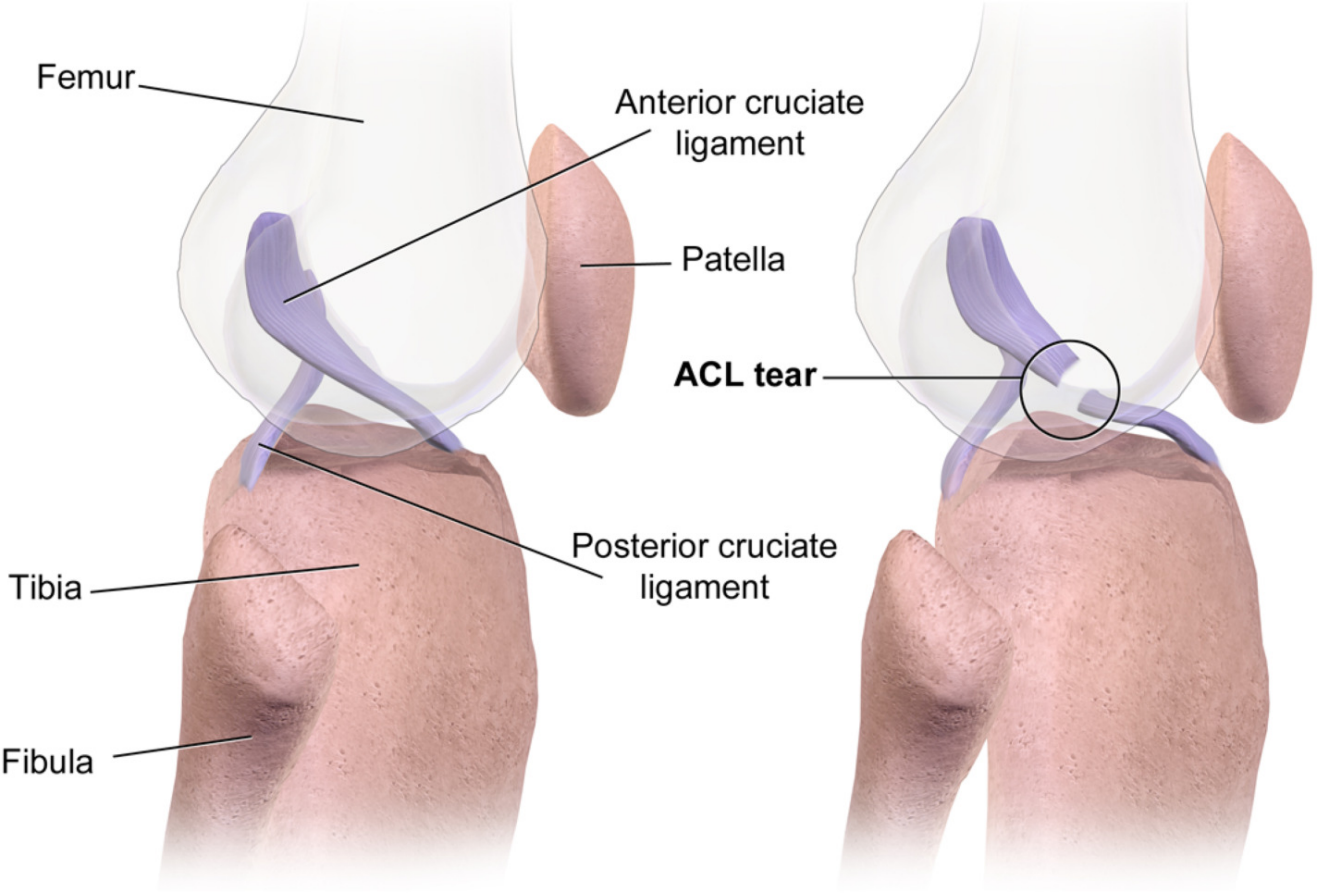
ACL tear. Sourced from Wikimedia Commons. Author: BruceBlaus https://commons.wikimedia.org/wiki/File:ACL_Tear.png. This work is free and may be used by anyone for any purpose. This file is licensed under the Creative Commons Attribution-Share Alike 4.0 International license.

The ACL of the knee is the most widely used and well-researched area of all-inside reconstruction techniques (Figure 2). From a technical point of view, all-inside ACL reconstruction has gone through a process from conceptualization to improvement and maturity. Currently, the standard all-inside ACL reconstruction utilizes a four-stranded folded semitendinosus tendon graft, which is approximately 60–80 mm in length and usually 8–9 mm in diameter to ensure adequate mechanical strength (30). The procedure is performed via two standard arthroscopic portals (anteromedial and anterolateral) with an additional small medial accessory incision. A retrograde drill first creates a 20–25 mm deep femoral socket in the femoral condyle, followed by a 15–20 mm deep tibial socket in the tibial plateau (31). The prepared graft is then sequentially pulled into the tibial and femoral sockets and fixed using cortical button suspension devices on the anteromedial tibial cortex and lateral femoral cortex, securing both ends within the sockets for anatomical reduction and tension balance. This “cortical-preserving” suspension design avoids distal tibial cortical perforation, maintains the integrity of the anteromedial tibial cortex, and promotes socket healing and stability. Research demonstrates that all-inside ACL reconstruction preserves bone mass in the distal femur and proximal tibia while achieving initial stability comparable to traditional techniques. Müller et al.'s study compared 2-year outcomes in patients with proximal ACL tears treated by *in situ* repair reinforced with InternalBrace vs. age- and sex-matched traditional hamstring autograft reconstruction and healthy controls (32). Both surgical approaches showed no significant differences in all subjective scores (mean IKDC ≈89; KOOS subdomains 80–90), achieving good to excellent levels. Rolimeter side-to-side differences were <3 mm, and isokinetic flexion/extension peak torque, H/Q ratios, and limb symmetry index (LSI ≈95%) showed no statistical differences between groups. However, all-inside reconstruction averaged 81 min in operative time, significantly shorter than traditional reconstruction. For strictly selected proximal ACL tear patients, all-inside reconstruction achieved comparable efficacy to traditional reconstruction in subjective, clinical, and functional outcomes within 2 years postoperatively while reducing operative time, suggesting its viability as an effective alternative for early ligament preservation. Nonetheless, optimal surgical timing is critical. Wenning et al. analyzed 444 isolated ACL rupture cases reconstructed with ipsilateral hamstring autografts, with preoperative and 5–7-month postoperative concentric isokinetic strength testing at 60°/s (33). Results showed that peak extension strength of the reconstructed knee decreased with surgical delay, and similar strength decline was observed preoperatively in the contralateral healthy limb. After adjusting for preoperative levels via covariance analysis, the early-surgery group exhibited significantly superior extension strength vs. delayed (*p* = 0.001) and chronic groups (*p* = 0.005), with flexion strength also outperforming delayed (*p* = 0.02) and chronic groups (*p* < 0.001). The hamstring-to-quadriceps ratio (H/Q) in the injured limb was overall higher than in the healthy limb and increased with surgical delay and postoperative follow-up, indicating progressive strength imbalance. To achieve ideal muscle strength at six months and facilitate return to sport, ACL reconstruction should ideally be completed within 12 weeks post-injury. Surgical delay not only weakens quadriceps and hamstring strength in the reconstructed limb but also affects the healthy limb, leading to bilateral thigh muscle decline. A retrospective analysis of 1,317 ACL reconstruction patients divided by injury-to-surgery interval (<3 months, 3–6 months, 6–12 months, >12 months) assessed meniscal injuries and repairability arthroscopically per ISAKOS classification (34). Univariate and multivariate analyses incorporated Tegner activity level, age, BMI, and sex. Results showed that surgical delay exceeding 12 months significantly increased medial meniscus (MM) injury incidence (*p* < 0.001), while delays within 3 or 6 months had no significant impact. Delay duration did not affect lateral meniscus (LM) injury rates. Each unit increase in Tegner level reduced MM injury risk by 10% (*p* = 0.020). Age >30 years and male sex were associated with higher MM injury risk; age >30 years also reduced MM tear repairability (*p* < 0.001), while male sex increased LM injury rate (*p* = 0.001). Delaying ACL reconstruction beyond one year post-injury significantly elevates MM injury risk without reducing repairability. High activity levels mitigate MM injury, whereas advanced age and male sex are independent risk factors for irreparable MM tears. To reduce MM injury risk, ACLR is recommended within 12 months post-injury. All-inside techniques are feasible and safe even in adolescent populations. In a multicenter retrospective study by Insam et al., 55 patients (mean age 8–24 years) undergoing combined ACL and anterolateral ligament (ALL) reconstruction were followed for 12.7 ± 1.5 months (35). Femoral ALL fixation used interference screws in 29 cases and SwiveLock anchors in 26 cases. Outcomes included instrumented anterior-posterior translation and rotational stability measurements, plus subjective Lysholm, Activity Rating Scale, Tegner, and VAS pain scores. Overall, mean anterior translation of reconstructed knees showed no significant difference vs. contralateral healthy knees. Internal rotation drawer test revealed a side-to-side difference of 3.3 ± 1.1 mm (vs. healthy side: 2.7 ± 1.0 mm; *p* = 0.0014), with no other significant translation or rotation differences. Lateral knee pain scores averaged only 1.1, and other patient-reported metrics remained comparable pre- to post-injury, indicating functional recovery to near-preinjury levels. Combined ACL + ALL reconstruction restored near-normal anteroposterior and rotational stability short-term without adverse clinical effects (e.g., incisional pain or excessive lateral knee stress), offering a safe and effective early treatment option for ACL tears with ALL injury. Mercurio et al.'s meta-analysis of 16 studies (2,329 patients: 1,116 combined ACL + ALL reconstruction. 1,213 isolated ACL reconstruction) found no significant differences in functional scores (Tegner, Lysholm, IKDC) (36). However, KT-1000 testing showed significantly greater residual anterior laxity in isolated ACL reconstruction (mean difference −0.44 mm). Conversely, combined reconstruction had significantly lower graft failure rates (*P* = 0.008), higher return-to-sport (RTS) rates, and improved graft survival (OR 2.94; *P* < 0.001). While subjective functional outcomes were comparable, combined ACL + ALL reconstruction significantly reduced postoperative knee laxity, decreased graft failure, and enhanced RTS and graft survival rates, providing robust evidence for improved long-term stability and athletic recovery.

All-inside ACL reconstruction has been shown to provide stability and functional recovery equivalent to the classic technique, while patients have less early pain and faster recovery, which is of great value in a population that needs to return to sport quickly. Table 1 summarizes the main differences in surgical approach and clinical performance of the all-inside technique vs. the conventional ACL reconstruction style.

**Table 1:** Comparison of conventional ACL reconstruction with all-inside ACL reconstruction.

Item	Traditional ACL reconstruction	All-inside ACL reconstruction	Long-term outcomes/evidence
Surgical incision	Requires full-length tibial tunnel → larger skin incision	Uses arthroscopic portals + one FlipCutter stab incision	Comparable anterior knee pain at >2 years; slightly lower early VAS in all-inside (Level II)
Tunnel preparation	Full femoral + tibial tunnels breach cortex	Blind femoral & tibial sockets preserve cortex	5-years tunnel widening 9%–15% vs 3%–7% (Level II)
Graft source	Two hamstrings or BPTB graft; greater donor-site morbidity	Quadrupled semitendinosus; gracilis spared	Hamstring strength deficit 10%-15% vs <5% at 2 years (Level II)
Fixation	Interference screw (tibia) ± screw/button (femur)	Cortical suspensory button + adjustable loop (both ends)	No difference in side-to-side KT-1,000 (2–3 mm) at ≥5 years (Level I)
Post-op pain	Screw irritation + BPTB harvest may cause anterior knee pain	Preserves cortex & periosteum → less pain, lower donor-site discomfort	Early VAS ↓20%-30% with all-inside; no long-term difference (Level II)
Rehabilitation	Pain & weakness may delay early rehab	Less pain enables accelerated protocols	Return-to-sport 9–12 months vs 7–9 months; not statistically significant in meta-analysis (Level II)
Bone preservation & revision	More bone loss; revision often 2-stage with bone grafting	Preserves bone stock; single-stage revision feasible	Single-stage feasible in 85% all-inside revisions vs <60% traditional (Level II)

Evidence grading is based on the 2011 OCEBM hierarchy: Level I = systematic review or multicenter RCT; Level II = single RCT or large prospective cohort; Level III = retrospective cohort or registry-based study.

### 4.2 PCL all-inside reconstruction

The anatomically concealed position of the PCL and the complexity of the posteromedial structures pose significant challenges for traditional PCL reconstruction. All-inside techniques offer a novel solution. All-inside PCL reconstruction typically follows the same principles as ACL reconstruction. Three to four arthroscopic portals including standard anterolateral and anteromedial portals plus posteromedial (and occasionally posterolateral) portals are used to create blind-ended sockets in the medial femoral condyle and the posterior tibial plateau, after which the graft is secured with adjustable suspensory fixation devices. A single 30° arthroscope provides adequate visualization for both femoral and tibial work. When needed, intraoperative fluoroscopic guidance may be employed to ensure precise placement of the posterior tibial socket (37, 38). Buranapuntaruk et al. studied 24 patients with multiligament knee injuries undergoing PCL reconstruction (PCLR): 11 with conventional techniques (CON-PCLR) and 13 with all-inside methods (AI-PCLR). At ≥2-year follow-up, kneeling stress radiographs showed side-to-side differences of ∼7.5 mm (CON-PCLR) vs. ∼5.8 mm (AI-PCLR) (*P* = 0.38). IKDC (68.9 vs. 73.9, *P* = 0.37), Lysholm (89.1 vs. 94.1, *P* = 0.42), and Tegner scores (6.0 vs. 6.4, *P* = 0.68) showed no significant differences. Complication rates were low. Results indicate comparable stability and patient satisfaction between AI-PCLR and CON-PCLR at ≥2 years (39). Similarly, Winkler et al. compared 169 isolated ACL reconstructions (ACL-R), 192 isolated PCL-R, and 203 combined ACL/PCL-R patients using KOOS questionnaires preoperatively and at 1/2 years postoperatively. All groups showed significant improvements, particularly in “Sport/Rec” and “QoL” subscales. From baseline to 2 years, ACL-R improved by ∼26 and ∼28 points, PCL-R by ∼20 and ∼23 points, and ACL/PCL-R by ∼19 and ∼21 points in these domains, respectively. These clinically meaningful gains highlight the importance of knee stability for sports recovery (40). Brossard et al. found that all-inside PCL reconstruction using autologous quadriceps tendon grafts, the prepared graft had a total length of 80 mm and a mid-segment diameter of 10 mm. The femoral and tibial sides were fixed using standard and extra-large adjustable cortical buttons, respectively. During surgery, posterior structures were exposed through 3–4 arthroscopic channels, including anterolateral, anteromedial, posteromedial, and transseptal approaches. The tibial blind tunnel was positioned at the posterior third of the tibial condyle using a 2.4 mm guide wire under fluoroscopic guidance, with a depth of 35–45 mm and a bone bridge of ≥7 mm preserved. The femoral blind tunnel is drilled to a depth of 25 mm at the medial condyle. This blind tunnel and adjustable suspension device not only maximize cortical bone preservation but also precisely control the length and diameter of the graft within the tunnel, effectively reducing “killer turn” stress and enhancing fixation reliability (41). Hoogeslag and colleagues found that by using the double-bundle all-intside PCL reconstruction technique to harvest two autologous hamstring tendons, the total length of the quadrupled graft can reach approximately 80 mm, with a diameter of 8–10 mm (42). Two femoral blind sockets and one tibial blind socket are drilled on the medial condyle using an external-internal drill, in conjunction with an adjustable suspension fixation device. the grafts were first independently fixed and tension-adjusted in their respective corresponding flexion-extension angles within the femoral blind sockets, followed by final fixation within a single tibial blind socket. This method avoids the stress concentration and graft “bottom-out” risks associated with the traditional “killer-turn” technique, while maximizing the preservation of cortical bone and soft tissue, reducing bone loss and complications. Additionally, a multicenter retrospective study by Chahla et al. comparing the efficacy of double-bundle and single-bundle PCL reconstruction in 441 cases confirmed that double-bundle reconstruction offers advantages in terms of objective stability and patient functional scores (43). Early PCL reconstruction is critical. A retrospective study of 148 primary multiligament PCL reconstructions found that delayed surgery (>12 weeks post-injury, *n* = 91) vs. early surgery (<12 weeks, *n* = 57) resulted in: Higher rates of concurrent LCL/PLC reconstruction (60% vs. 40%, *p* = 0.02). More frequent cartilage lesions (46% vs. 24%, *p* = 0.01), especially lateral (19% vs. 5%, *p* = 0.04) and medial femoral condyles (34% vs. 11%, *p* = 0.005). These findings support reconstruction within 12 weeks to preserve joint integrity (44). PCL reconstruction outcomes may lag behind ACL reconstruction. Mestriner et al. followed patients for 2 years post-arthroscopic autograft reconstruction. While both PCL and ACL groups showed significant improvements from baseline (*p* < 0.001), the PCL group had lower objective IKDC scores than the ACL group (*p* < 0.001), though Lysholm (*p* = 0.052), clinical tests (*p* = 0.058), and KT-1000 results (*p* = 0.129) were comparable (45). A systematic review of 19 ACL-PCL reconstruction studies noted that 90% tensioned/fixed the PCL before the ACL. PCL fixation angles were typically 70°–90° flexion, while ACL fixation ranged from 0° to 70° (46). Despite technical variations, patient-reported outcomes were similar across studies All-inside PCL reconstruction yields satisfactory results even in adolescents. Razi et al. studied 55 PCL rupture patients (mean age ∼20 years). “Single-tibial/double-femoral tunnel” was the most common technique. Cincinnati scores improved from ∼35.9 preoperatively to ∼79.5 postoperatively (*p* < 0.001). Full knee motion increased from 29.1% to 92.7%. Complications included stiffness (3 cases), DVT (1), and infection (3). Isolated PCL injuries had higher preoperative CKRS (*p* = 0.006) and lower posterior drawer grades (2.76 vs. 3.10, *p* = 0.042) than multiligament injuries. Modern PCL reconstruction significantly restores function and mobility, supporting surgical intervention even for isolated ruptures to prevent long-term deficits and degeneration (47). In conclusion, the differences in clinical performance and technical difficulty between ACL and PCL (Table 2).

**Table 2:** Clinical outcomes and technical considerations of All-inside ACL vs PCL reconstruction.

Parameter	All-inside ACL	All-inside PCL	Principal technical challenge
IKDC (2 years, median)	85–92	70–80	Femoral socket angle & graft passage limited by posteromedial structures
AP/PD laxity (instrumented)	KT-1000 1.2–2.5 mm	Posterior drawer 2.5–4.0 mm	Avoiding popliteal neurovascular injury during tibial socket creation
Lysholm score	90 ± 4	86 ± 5	
Tunnel widening ≥10 mm	3%–7%	5%–10%	PCL tibial socket is shorter, prone to “killer-turn” abrasion
Graft failure	<5%	5%–8%	
Preferred graft	Quadrupled semitendinosus ± gracilis	Quad-strand tibialis anterior or peroneus longus	PCL requires thicker/longer graft

Values for isolated single-bundle PCL; multiligament cohorts report posterior laxity 5.8–7.5 mm. Imaging studies at 6–12 months show 5%–10% of tibial sockets enlarging >10 mm.

## 5 Graft selection and fixation methods

### 5.1 Graft selection

The type of graft used in all-inside reconstruction affects postoperative biomechanical performance and healing. Common options include autografts (such as hamstring tendon, patellar tendon, quadriceps tendon) and allografts. Hamstring tendons are the most commonly used in all-inside ACL reconstruction due to their relatively simple harvesting and fewer complications. Specifically, all-inside techniques typically require only the semitendinosus tendon, which is folded into four strands to achieve the required diameter, avoiding the use of the gracilis tendon and thus reducing hamstring injury. Bone-patellar tendon-bone (BTB) grafts are less commonly used in all-inside techniques because the bone blocks at both ends cannot be fully fixed within blind tunnels, requiring modified techniques or additional small incisions to secure the bone blocks (48). However, BTB has unique advantages, including faster bone-to-bone healing and high initial stability. Shichman et al. studied 286 patients undergoing BTB autograft ACL reconstruction, dividing them into a bone grafting group (*n* = 198) and a non-grafting group (*n* = 88) based on whether the patellar donor-site defect was filled with bone substitute (49). At the final follow-up, the bone grafting group had a significantly lower incidence of anterior knee pain (*p* = 0.004), lower pain intensity (VAS score, *p* = 0.004), and a significantly reduced overall complication rate (*p* = 0.027). No significant differences were found in Lysholm, IKDC, or SF-12 scores between the two groups. The study concluded that filling the patellar donor-site defect with bone substitute in BTB autograft reconstruction can effectively reduce the incidence and severity of postoperative anterior knee pain, but larger randomized controlled trials are needed to further validate long-term benefits. Similarly, another study reviewed 7 patients with chronic patellar tendon rupture treated with contralateral BTB autograft reconstruction. Preoperative radiological evaluation showed abnormal patellar height (Caton-Deschamps index) in 86% of patients, with a mean of 1.5 (50). Postoperatively, this improved to 1.2 (*p* = 0.0136), indicating normalization of patellar height. Functional scores improved significantly: IKDC from 45.5 to 64.5 (*p* = 0.0001), Lysholm from 45.4 to 79.0 (*p* = 0.0001), and Tegner activity level from 1 to 4 (*p* = 0.0001). The preoperative median activity level was 6. All patients had persistent quadriceps atrophy on the affected side, with a thigh circumference difference of 3.6 cm, and no surgical complications were reported. Contralateral BTB transplantation for chronic patellar tendon rupture can safely and effectively restore patellar height and significantly improve subjective and objective postoperative function, though full recovery to pre-injury levels was not achieved. Additionally, studies using quadriceps tendon for all-inside ACL reconstruction found its stability comparable to BTB and hamstring autografts (51). Allografts are commonly used in multi-ligament injuries or revision surgeries and are also applied in all-inside techniques. A systematic review of all-inside PCL reconstruction found that over half of the literature favored allografts due to the need for long and thick grafts, which avoids excessive autograft harvesting. Allografts shorten surgical time and avoid donor-site morbidity but have slower healing and higher risks of infection and failure. Graft selection in all-inside reconstruction requires balancing donor-site conditions and biomechanical needs (52). For ACL, all-inside techniques prefer autologous semitendinosus tendon, supplemented with gracilis tendon or allografts if the semitendinosus is insufficient (53). Ensuring a graft diameter ≥8 mm is critical, as studies show a 6.8-fold increased failure risk with autografts <8 mm. For high-demand ligaments like PCL, allografts are a good option, but postoperative healing and re-rupture risks should be monitored. Rational graft selection and optimized configurations (e.g., four-strand folding, double-bundle reconstruction) can improve the success rate and long-term stability of all-inside reconstruction (54).

### 5.2 Comparison of fixation methods

Graft tunnel fixation is one of the core techniques in all-inside reconstruction, directly affecting initial stability and long-term bone-tendon healing. The main fixation methods are divided into two categories: aperture fixation and cortical suspensory fixation. In traditional ACL reconstruction, the tibial end often uses bioabsorbable or metal interference screws to wedge the tendon against the bone tunnel wall, achieving tight fixation at the tunnel opening (55). The femoral end may use a transverse EndoButton or interference screws. Aperture fixation's advantage is close contact between the graft and tunnel wall, with minimal initial displacement and high pullout stiffness, promoting bone-tendon healing (56). However, its drawbacks include potential communication between the bone tunnel and joint cavity, leading to synovial fluid infiltration and tunnel widening. Additionally, screws may cause stress concentration, increasing the risk of localized graft damage and tunnel expansion. In contrast, suspensory fixation uses a button plate and high-strength loop to suspend the graft outside the cortical bone, effectively “hanging” the tendon within the tunnel. Its advantages include preserving the integrity of the opposite cortical bone and allowing intraoperative tension adjustment in some designs. Biomechanical tests show that suspensory fixation often has higher ultimate load capacity, slightly greater tensile elasticity, and better toughness than interference screws. However, suspensory fixation has drawbacks, such as axial elastic elongation of the graft within the tunnel and lateral friction against the tunnel wall, which may lead to tunnel widening and graft micromotion, hindering bone-tendon healing. A study on quadriceps tendon autograft ACL reconstruction found that aperture fixation and suspensory fixation had similar short-term clinical outcomes, but differences existed in key metrics: suspensory fixation had a higher proportion of patients achieving IKDC A/B grades at both femoral and tibial ends (81.7% vs. 67.7%), with smaller anterior laxity differences (1.6 mm vs. 2.3 mm) (57). Current reports of graft failure are all associated with aperture fixation, with a failure rate of approximately 3.2%. Overall, regardless of fixation method, the short-term failure rate for quadriceps tendon grafts is around 3%, suggesting both methods are equally effective in functional recovery, but suspensory fixation may have slight advantages in knee stability and lower failure risk. Therefore, in all-inside reconstruction, most scholars prefer using cortical suspensory fixation at both ends to fully embody the concept of “cortical bone preservation” in all-inside techniques. It should be noted that the use of adjustable loops facilitates intraoperative adjustments but may introduce additional elongation risks. Related studies have reported that reconstructions using adjustable suspensory devices at both ends exhibit slightly inferior early stability compared to fixed-loop suspensory fixation, as adjustable loops may gradually elongate under cyclic loading. To address this issue, some medical scholars have proposed improvements. Kocabey and colleagues reported that replacing the femoral end with a fixed-loop button, making it non-adjustable, can effectively prevent postoperative loop loosening and enhance initial stability (58). Additionally, studies on improved graft-loop connection methods, such as the “buried-knot technique”, have shown through *in vitro* testing that they can significantly improve construct stability. In the field of all-inside reconstruction, suspensory fixation is currently the mainstream, but the selection and improvement of specific devices are still evolving. Rational application of fixation techniques can further optimize the biomechanical performance of all-inside reconstruction.

## 6 Imaging assessment and biomechanical studies

### 6.1 Imaging assessment

Imaging has an important role in the assessment of the efficacy and monitoring of complications of all-inside reconstruction. Conventional radiographs can be used to assess changes in the location and diameter of the bone tunnel: follow-up x-rays after all-inside reconstruction often show less enlargement of the diameter of the tibial bone tunnel compared to the conventional through-tunnel technique, which is thought to be related to cortical integrity and synovial fluid isolation (59, 60). In a randomized controlled study at 2 years postoperatively, radiographic measurements revealed that the tibial bone channel expanded only 1.1 mm on average in the all-inside-suspension button fixation group, compared with approximately 3.0 mm in the conventional interface screw fixation group, with better healing of the bone channel in the former group (61). Good healing of the bone groove is especially beneficial for patients who need a second surgery, which can reduce the need for bone grafting during revision, and MRI can visualize the signal of the graft in the bone groove and the surrounding bone reaction, which can help to determine the bone-tendon healing and the presence of bone tunnel cystic degeneration. In general, the gradual re-vascularization of the graft and the absence of significant cystic changes in the bone surrounding the bone tunnel are indicative of good healing when seen on MRI at about 6 months postoperatively (62). Some studies have compared the MRI features after all-inside vs. conventional ACL reconstruction and found that the all-inside group had reduced signal intensity in the middle portion of the graft (suggesting a better blood supply) and a less severe edematous reaction around the tibial malleolus. In addition, imaging evaluation includes secondary arthroscopic exploration: in some cases with unexplained symptoms, the graft tension, wear, and intraosseous groove can be visualized by secondary arthroscopy (63). A “bottoming-out” phenomenon due to excessive length of the original graft, despite good continuity, has been reported in revision 1 year after total ACL reconstruction, suggesting that intraoperative groove depth measurements should be more precise to avoid graft laxity (64). Overall, routine follow-up x-rays and MRIs can help the surgeon to monitor bone channel changes and graft status in allograft reconstruction, which is important for early detection and management of complications such as bone channel capsulorrhaphy and graft breakage. Current imaging studies support that the all-inside technique is effective in reducing bone channel expansion.

### 6.2 Biomechanical studies

A large number of biomechanical experiments have provided theoretical support and directions for improvement of the all-inside reconstruction technique. In terms of knee stability, *ex vivo* and *in vivo* studies have generally confirmed that all-inside ACL reconstruction is no less effective than traditional techniques in terms of anatomical localization and mechanical properties (65). A meta-analysis of 5 RCTs and 4 cohort studies showed that there was no significant difference between all-inside cortical suspension fixation and conventional all-tunnel fixation in terms of anterior knee stability, rotational stability, and other clinical indicators. Mayr et al. found that the all-inside group was comparable to the conventional group in objective stability tests such as tibial anterior displacement and Lachman's test in a comparative study, but that the all-inside group was comparable to the conventional group due to the larger diameter of the grafts in terms of stress distribution (66). The larger diameter was closer to the normal knee joint in terms of stress distribution. Biomechanical testing also provided guidance on the details of the different allograft techniques. In terms of graft preparation, Tiefenboeck and colleagues and others compared suspension fixation of grafts with nodes buried (buried-knot) vs. conventional knotting and showed that the buried-knot technique significantly improved the durability of the graft-collar connection (67). In terms of fixation devices, one experiment tested the biomechanics of continuous loop-suspended grafts in different fixation positions in a porcine bone model and found that placing the fixation buttons on the lateral cortex avoided screw fixation-induced graft lengthening and that the initial stiffness of suspension fixation performed well in terms of fatigue resistance. Another simulation study examined the effect of different drilling angles of the allograft on the biomechanics of the knee, and the results showed that drilling the femoral groove using the anteromedial approach was more in line with the anatomical course of the ACL, and better restored the stability of the knee at different flexion angles. However, the anteromedial technique is demanding for the operator and may increase the risk of positioning errors and complications (68, 69). Therefore, some researchers have suggested that the lateral femoral approach should be used in specific cases to improve surgical reproducibility and reduce the chance of injury to the proprioceptors of the ACL stump. In addition, computerized finite element analysis has been applied to the biomechanical study of all-inside reconstruction. Some models showed that under the same load, the bone stress distribution of all-inside suspension fixation was more dispersed and the maximum stress was lower than that of the screw fixation model, which theoretically supports the protective effect of suspension fixation on the bone tunnel. Overall, biomechanical studies have provided data to support the safety and effectiveness of all-inside reconstruction and have pointed out directions for improvement. For example, optimization of the bone tunnel length and diameter ratio based on experimental results, and improvement of the suspension device to prevent micromotion are continuously improving the performance of all-inside reconstruction.

## 7 Postoperative rehabilitation

The principles of postoperative rehabilitation after all-inside reconstruction are broadly similar to those of conventional ligament reconstruction, i.e., gradual restoration of joint mobility and muscle strength while protecting the graft. However, due to the minimally invasive advantages of the all-inner technique, patients can often be more active in early functional exercises. Many factors influence rehabilitation after ligament reconstruction, including the initial strength of the graft, the rate of bone-tendon healing, changes in the mechanical properties of the graft material over time, and the stress loads applied to the graft during rehabilitation (70). All-inside reconstruction has some favorable conditions in these areas: first, using only the semitendinosus tendon and preserving the thin femoral muscle reduces the weakening of the knee flexor muscle groups, which contributes to a faster recovery of muscle strength after surgery. Studies have shown that patients who retained the femoral thinning muscle had significantly better postoperative flexion strength, especially at high flexion angles, than those who had both hamstrings removed (71). Second, because the bone cortex was not penetrated, postoperative proximal tibial pain and weight-bearing discomfort were less severe, and patients dared to go down to weight-bearing earlier. This created conditions for early rehabilitation training (e.g., proprioception and balance training) to be carried out. In addition, progressive joint mobilization exercises can be started on the same day after ACL all-inside reconstruction, with the goal of preventing joint stiffness in the fully extended position (72). Quadriceps isometric contraction exercises can be performed and weight-bearing walking can be performed on the 23rd postoperative day under the protection of a brace, as long as the patient's pain is tolerated. In most cases, unassisted full range of motion and light exercise such as jogging can be achieved 6–8 weeks after surgery. In contrast to conventional ACL reconstruction where the stability of screw fixation in the bone tunnel is a concern, suspension fixation for all-inside reconstruction performs well under cyclic loading and permits a slightly accelerated rehabilitation pace (73). Of course, the rehabilitation process should still be individualized. Especially during the first 12 weeks before bone-tendon healing is complete, excessive weight-bearing knee flexion (>90°) should be avoided to avoid excessive shear stress on the graft. In patients with all-inside reconstruction of the PCL, posterior tibial transfer forces should be avoided in the early postoperative period because of weak posterior stabilization, such as squatting, which should be delayed, and post-padding of the knee to limit hyperextension (74). After SCR of the shoulder, a 6-week cast support is needed to protect the graft from healing, and then mobility and rotator cuff muscle strength training are gradually increased. Rehabilitation after all-inside reconstruction requires a balance between protecting the graft and restoring function. The good news is that patients experience less postoperative pain and muscle inhibition due to minimally invasive procedures and limited graft acquisition, which leads to better compliance and results in early rehabilitation (75). A study of all-inside ACL reconstruction reported a higher percentage of patients with excellent recovery of Lysholm scores in the all-inside group than in the conventional group at 3 months postoperatively, which the authors attributed to the fact that the all-inside technique prompted patients to enter into effective functional training earlier (76, 77). With strict precautions against re-injury, taking full advantage of the early pain relief and mobility provided by the all-inside technique and developing an active and rational rehabilitation program will help patients return to daily life and competitive sports as soon as possible.

## 8 Complications and their prevention and control

Any surgical procedure carries some risk, and all-inside reconstruction is no exception, but its complication rate is generally low. Common complications include re-breakage or laxity of the graft, enlargement of the bone tunnel, and damage to cartilage or adjacent structures. A series of preventive and remedial measures have been summarized by clinical and research workers to address these problems (Table 3).

**Table 3:** Common complications of All-inside techniques and their management.

Type of complication	Affected joint(s)	Reported incidence	Typical management strategies
Graft laxity/rupture	Knee, shoulder, ankle	1.4%–3.5% (knee ACL) ≤5% (shoulder SCR)	• Mild laxity: brace + targeted rehab • Complete failure: single-stage or two-stage revision
Tunnel widening	Knee	3%–7% with ≥10 mm expansion	• Observation if asymptomatic • Bone graft or dowel packing when symptomatic or >10 mm
Hardware slippage/loosening	Knee, shoulder	No robust incidence; mainly case reports	• Re-tension or exchange fixation device • Add back-up knots or convert to fixed-loop button
Iatrogenic chondral injury	Knee, hip	<1% (arthroscopic detection)	• Arthroscopic debridement or microfracture • Avoid deep guide-pin penetration intra-op
Deep or superficial infection	Knee, shoulder	0.2%–0.6%	• Prompt irrigation + systemic antibiotics • Remove graft/hardware if uncontrolled
Neurovascular injury	Knee, hip	<0.1% (isolated case series)	• Maintain knee flexion >90° during drilling • Electrophysiologic follow-up or surgical decompression when needed

### 8.1 Graft laxity and breakage

Some studies have noted that a slightly higher rate of graft laxity or failure may occur with all-inside ACL reconstruction, but there is no statistically significant difference and the clinical significance is questionable. To minimize the risk of graft laxity, it is important to ensure that the depth of the bone trough is matched to the length of the graft intraoperatively to avoid buttons that are already against the bone cortex before the graft is tightened (the so-called “graft bottoming out” phenomenon). Adjustments can be made by accurately measuring the length of the bone channel and reserving excess graft length for the femoral end (to ensure that the tibial end does not bottom out) (78). In addition, initial graft tension is tightly controlled during reconstruction, and a stretching device is applied to pre-tension the graft prior to femoral fixation to minimize the potential for postoperative laxity. Adjustable suspension fixation devices have an initial risk of ring buckle slippage, which can be prevented from lengthening under weight-bearing by locking it with a knot or by switching to a fixed long loop device. The above improvements have been shown to improve early graft stability. Regarding graft re-fracture, the incidence of allografts is comparable to that of the conventional technique, occurring mostly within 1–2 years postoperatively in a high-risk active population (79). The key to prevention is to avoid early strenuous exercise during the postoperative rehabilitation phase and to ensure that the graft diameter is adequate (80). Graft survival in all-inside reconstruction is as reliable as in conventional reconstruction, provided that the technique is performed correctly and postoperative management is improved. In all-inside ACL reconstruction, a quadrupled semitendinosus autograft (ST4) is routinely chosen as the primary graft. When the final graft diameter is below 8 mm, the risk of graft failure rises markedly, particularly in high-demand athletes (81, 82). If intra-operative measurement shows an ST4 diameter smaller than 8 mm, the surgeon can enlarge it by adding the gracilis tendon to create a five- or six-strand autograft or by suturing a small strip of soft-tissue allograft around the autograft bundle, ensuring a final diameter of at least 8 mm (83, 84, 85). In situations such as multiligament injury, revision surgery, or when the patient declines additional autograft harvest, a full soft-tissue allograft measuring 8 mm or more can serve as the primary graft (86, 87). When these diameter-based strategies are applied, graft survival with the all-inside technique has been shown to equal that of conventional full-tunnel reconstruction.

### 8.2 Bone tunnel enlargement

Bone tunnel enlargement is often caused by graft micromotion or synovial fluid infiltration, which can lead to revision difficulties. With the all-inside technique, the rate of bone tunnel enlargement is significantly reduced due to the closure of the bone channel and the isolation of the synovial fluid. However, enlargement of the bone tunnel may still occur if fixation is not stable, especially if the adjustable ring is progressively loosened or if there is a significant wiper effect of the graft. Preventive measures include intraoperatively ensuring that the graft is tightly attached to the bone channel in all radial directions and, if necessary, filling the gap with a biocollagen or absorbable spacer in the inner portion of the bone channel to minimize graft wobble and synovial fluid entry (88). Loosening of the fixation device is another predisposing factor, and a product that has been shown to be stable should be used and properly locked according to the instructions. As mentioned previously, using a fixation long loop button at the femoral end whenever possible will minimize enlargement of the bone socket due to loosening of the adjustable ring. Avoiding excessive weight bearing and significant flexion and extension activities in the early postoperative period will also contribute to good healing of the socket (89). During imaging follow-up, one should be alert to the presence of translucent areas or cystic shadows around the bone groove, and once detected, implantation of bone clay or local compression bone grafting can be considered to promote healing.

### 8.3 Difficulty in revision

Conventional ACL reconstruction often encounters the problem of oversized or overlapping tunnels in the original bone. All-inside reconstruction reduces the difficulty of revision by preserving the amount of bone and keeping the tunnels small. However, if the initial all-inside reconstruction is performed with improper positioning of the bone tunnel, a new bone tunnel will still need to be re-established during revision, which may intersect with the original bone tunnel (90). The key to avoiding this problem is to accurately localize the anatomical position of the bone trough during the initial surgery and try to align it with the natural ACL attachment area, so as to avoid the difficulty of future revision due to incorrect position. In addition, the surgeon may consider using a through-bone tunnel with bone grafting to fill and reshape the original bone channel during revision, and then re-establishing it using the all-inside technique (91). This “one-stage revision” is easier to accomplish with the all-inside technique because the original bone channel is smaller in diameter, making bone grafting easier to accomplish. Clinical reports have shown that allograft ACL reconstruction without staged bone grafting has a higher success rate because of the good condition of the bone socket and the wide choice of grafts.

### 8.4 Other surgical complications

The operative specificity of the all-inside technique also has some potential risks that need to be guarded against by the operator. When preparing the femoral bone groove via the anteromedial approach, if the angle is skewed, structures such as the common peroneal nerve around the lateral femoral condyle may be injured. In this regard, experience suggests that intraoperative knee flexion should be strictly maintained past 90° and that the guide pin should not be drilled too deeply into the lateral femoral skin to ensure that it is away from the neurovascular bundle. In addition, when using a rotary drill, care should be taken to protect the original residual ligamentous tissue, especially if it is desirable to preserve a portion of the ACL bundle (92). If proprioceptive function of the ligament remnant needs to be preserved, a flip drill technique that would disrupt the remnant is not recommended, and the groove can instead be drilled through a small lateral femoral incision. Arthroscopic access should also avoid multiple punctures to the same location to avoid soft tissue “tunneling” and fluid leakage (93). Complications of SCR of the shoulder are mainly re-breaking of the patch and loosening of the fixation anchors, which should be prevented by making sure that the patch is properly sized and the suture tension is evenly distributed, and by instructing the patient to strictly follow the rehabilitation limitations, avoiding premature and excessive shoulder abduction and supination (94). In hip labral reconstruction, it is important to avoid deep placement of labral anchors to the articular cartilage surface, which can be confirmed by direct arthroscopic observation of the depth of the anchors or by x-ray fluoroscopy (95). In general, most of the complications associated with All-inside reconstruction can be prevented by standardized practice and postoperative management. In case of problems, such as mild enlargement of the bone groove or laxity of the graft, close observation and rehabilitation can be supplemented; in severe cases (e.g., fracture of the graft), revision surgery can be performed in a timely manner.

In summary, the all-inside reconstruction technique has shown relative advantages in reducing complications, such as tibial plateau fracture and infection with a very low incidence. However, the surgeon still needs to be skillful in the technique and consider precautions in advance to ensure the safety and success of each surgery. With advances in instrumentation and materials, the safety of the all-inside technique is expected to improve further, bringing patients greater peace of mind in their treatment choices. Here, we comprehensively summarize the clinical trials related to all-inside reconstruction techniques that currently exist (Table 4).

**Table 4:** Clinical trials related to all-inside reconstruction techniques.

NCT number	Study title	Study status	Conditions	Interventions	Phases
NCT06899659	Evaluation on Safety and Effectiveness of the An All-inside, All-suture Meniscal Repair Device	Completed	Meniscal Tears	DEVICE: JuggerStitch? Meniscal Repair Device| DEVICE: Fast-Fix 360 Meniscal Repair System	
NCT05491564	SoftStitch? for All-Inside Meniscal Repair: Comparative Analysis of Patient Reported Outcome Measures	Unknown	Meniscus Tear	DEVICE: SoftStitch?	
NCT05574946	Comparison of Two ACL Reconstruction Techniques: All-inside Versus Complete Tibial Tunnel Technique	Completed	Anterior Cruciate Ligament Tear	PROCEDURE: Anterior Cruciate Ligament Reconstruction	
NCT06067945	Comparison of Marginal Fit and Internal Adaptation in All-Metal Crowns: an *in vitro* Experimental Trial	Recruiting	Evaluation of Marginal and Internal Fit of Metal Crowns	OTHER: microleakage	
NCT02052856	Anterior Cruciate Ligament (ACL) Tunnel Widening Comparing All-inside and Interference Screw Fixation Technique	Completed	Anterior Cruciate Ligament Injury|Knee Injury|ACL Injury		
NCT01448278	Pain Evaluation After Anterior Cruciate Ligament (ACL) Ligamentoplasty	Completed	Arthroscopic Anterior Cruciate Ligament Reconstruction	PROCEDURE: “All-inside”|PROCEDURE: Classical technique	PHASE4
NCT05580133	All-Inside Single-Bundle for Anterior Cruciate Ligament Reconstruction With Full Thickness of the Peroneus Longus Tendon Compared to the Six-strand-hamstring Autograft (ACL)	Unknown	Anterior Cruciate Ligament Injuries	PROCEDURE: anterior cruciate ligament reconstruction	
NCT04903106	Safety and Performance Study of the FAST-FIX FLEX System for Meniscal Repairs and Meniscal Transplantations	Completed	Meniscus tear	DEVICE: FAST-FIX FLEX Meniscal Repair System|DEVICE: FAST-FIX FLEX Meniscal Repair System	

## 9 Limitations and prospects

### 9.1 Limitations

The emergence and maturation of the all-inside reconstruction technique has brought many benefits to orthopedic arthroscopic surgery. The all-inside technique has achieved impressive results in the field of ligament reconstruction in terms of both anatomical and clinical outcomes. It achieves approximate or even better stability reconstruction through a minimally invasive approach compared to conventional reconstruction, while significantly reducing surgical trauma and postoperative pain. Patients have a faster functional recovery, good early regain of knee mobility and muscle strength, and a high degree of subjective satisfaction. These advantages make the all-inside technique particularly suitable for competitive athletes and young, highly active patients, and provide a safer solution for multiple ligament injuries and adolescent ligament injuries. However, all-inside reconstruction is not perfect and there are still some limitations and challenges.

First, there is a steep learning curve: the technique requires the operator to be skilled in arthroscopic manipulation and spatial positioning, and precise control of the angle and depth of the bone groove is dependent on experience. Beginners often need some accumulation to achieve results on par with traditional procedures. For this reason, specialized training and simulation exercises are important, and mastery can be improved through cadaveric experiments. Multiple clinical and simulation studies have quantified the learning curve for all-inside ACL reconstruction. The largest analysis to date (a MOON registry subgroup of 742 cases) showed that after approximately 30 procedures, the average operative time fell from 97 ± 15 min to 78 ± 12 min, and the intra-operative complication rate declined to a level comparable with full-tunnel techniques (96). A prospective single-centre series of 60 cases demonstrated that by the 25th case, operative time, graft-tension accuracy, and socket-angle error had all plateaued, with deviations stabilising within ± 2 mm/°. Time-series analysis of 159 consecutive cases further indicated that graft re-tear risk drops markedly after 20–30 procedures and reaches its lowest point beyond 150 cases (97). Moreover, a randomised controlled trial showed that 10 virtual-reality sessions combined with 3 cadaveric dissections can raise a trainee's first independent all-inside ACL reconstruction performance to the level of a surgeon with 20 live cases, significantly shortening the learning curve. Collectively, these findings suggest that surgeons should complete at least 25–30 supervised cases, supplemented by VR or cadaver simulation, before performing all-inside ACL reconstruction independently to achieve operative efficiency and safety comparable to experienced surgeons (98).

Second, the controversy of proprioception and blood supply: whether the all-inside technique, which avoids bone tunnel penetration, affects the blood supply of the bone marrow cavity to promote the healing of the graft is still inconclusive. Some studies have suggested that closure of the bone tunnel may reduce bone marrow exudation and be detrimental to graft vascularization, but actual clinical results have not shown healing problems. On the other hand, the use of a flipper drill may remove mechanoreceptors from the original ligamentous stump, potentially weakening joint proprioception. Therefore, in cases where preservation of the ligament remnant is desired, a modified surgical approach may be considered to protect proprioceptive function. Preservation of the residual ACL/PCL stumps allows retention of mechanoreceptors such as Ruffini and Pacinian corpuscles at the ligament ends, providing a “hardware” foundation for early proprioceptive feedback in the graft. Subsequent revascularization and ingrowth of nerve fibers into the graft enable “rewiring”, progressively restoring joint position sense and kinesthesia. In a prospective cohort study, Drigny et al. enrolled 30 primary ACL reconstruction patients and 20 healthy controls (99). At 4 months postoperatively, proprioception was objectively evaluated using three metrics: joint position sense–initial detection (JPS-1), reposition accuracy (JPS-2), and threshold to detect passive motion (TTDPM). The surgical knees outperformed the contralateral knees in TTDPM (*p* = 0.008), while no significant differences were observed in JPS-1 and JPS-2 compared to controls, suggesting early compensatory recovery of kinesthesia but a delayed restoration of position sense. Notably, when the injury occurred on the non-dominant leg, JPS-2 errors significantly increased, indicating a dominance-related impact on reposition accuracy. The addition of an anterolateral extra-articular procedure did not significantly affect any proprioceptive indices. At 8 months, psychological readiness for return to sport was assessed using the ACL-RSI scale, revealing a moderate yet non-significant negative correlation with JPS-2 (r ≈ −0.38), implying a limited association between psychological status and position sense. Overall, kinesthesia may surpass the contralateral side as early as 4 months postoperatively, while position sense recovery is slower and influenced by leg dominance. Therefore, during rehabilitation, position sense training should be intensified for the non-dominant leg, and return-to-sport timing should be optimized through integrated psychological-functional assessment. In an anatomical study, Lin et al. cannulated the three main arteries in 14 fresh-frozen human knee specimens and injected gadolinium contrast to evaluate regional perfusion across the proximal, middle, and distal thirds of the native ACL (100). The proximal third received 56% ± 17% of total ligamental perfusion, significantly more than the middle (28% ± 15%, *p* = 0.007) and distal thirds (16% ± 16%, *p* = 0.001). Males exhibited higher perfusion in the proximal ACL (67% vs. 48%, *p* = 0.036). These findings provide imaging-based evidence for the concept of “vascular enrichment toward the femoral attachment” and support the intrinsic healing potential of proximal tears, emphasizing the importance of preserving the proximal remnant during surgery to facilitate revascularization. Additionally, Arai et al. evaluated 32 autograft ACLR patients at 3, 6, and 12 months postoperatively by measuring the signal-to-noise ratio (SNR) along the bone tunnel wall and determining the shortest distance from the tunnel surface to adjacent vascular networks (e.g., posterolateral tibial venous plexus and intramedullary tibial artery) (101). Sections within 5 mm of a vessel showed significant revascularization by 3 months (SNR increase of 43%, *p* < 0.01), whereas segments >10 mm from a vessel required up to 12 months to reach equivalent signal levels. For each additional millimeter of distance, signal recovery at the bone–tendon interface was delayed by approximately 0.7 months (R^2^ = 0.64). Multivariate regression confirmed vessel proximity as the strongest independent predictor of early osteointegration, while graft diameter and fixation technique showed no significant influence. These findings underscore the importance of optimizing bone tunnel geometry to minimize graft-to-vessel distance and suggest that adjunctive interventions such as PRP or BMAC application may be beneficial at poorly vascularized distal sites to enhance graft integration.

Third, improvement of fixation devices: the current adjustable suspension fixation is convenient, but there is a risk of loosening of the ring buckle; to solve this problem, a new generation of fixation devices (e.g., ring buckles with self-locking mechanisms, double-button fixation systems, etc.) is being developed to achieve both convenience and zero slip. In addition, the addition of adjuncts to suspension fixation (e.g., medial anchors, internal support straps) has been shown to help improve early stability and reduce graft loosening, and may become one of the standard features in the future. Recent years have witnessed the emergence of four “next-generation” fixation systems with notable breakthrough potential. Self-locking adjustable loops (e.g., LoopLoc®, TigerLoop) incorporate a ratcheted or serrated locking mechanism within the loop, permitting secondary tensioning and virtually eliminating loop slippage (102). Multicenter retrospective cohorts have confirmed excellent early stability and no significant tunnel widening within the first year compared with conventional adjustable loops; however, ≥5-year follow-up data and evidence of long-term bone-tunnel remodeling remain lacking. Double-button constructs (e.g., Button-Button, ZipLoop) deploy cortical buttons on both the femoral and tibial sides to create a truly “zero-slip” suspensory system. A randomized controlled trial (NCT05328544) is currently recruiting, and bench-top cyclic-loading tests already show reduced lengthening, but clinical failure modes and early loosening rates still need clarification. Bio-inductive scaffolds (BioBrace®) integrate a porous collagen-PLLA matrix into the adjustable loop, providing dual “structural + biologic” reinforcement of the graft–bone interface. A multicenter safety-and-performance study (NCT06948591, 2023–2028) is quantifying MRI-PDFF and T2-based healing signals, yet standardized imaging metrics for scaffold remodeling are still unavailable. Fully bioresorbable cortical buttons or magnesium-alloy fasteners employ PLLA or Mg-Zn-Ca alloys that degrade within 18–24 months and are replaced by new bone, aiming to eliminate tunnel abrasion and the need for hardware removal. Nevertheless, a 3-year RCT (NCT03529552) has not yet released final results, and concerns persist regarding late tunnel bone loss and screw-track osteolysis after degradation. Collectively, these devices tackle fixation challenges from the perspectives of “mechanical anti-slip with bidirectional locking”, “force-biology coupling”, and “complete material resorption”. While short-term biomechanics and safety have been preliminarily validated, definitive evidence for durability, MRI-based quantitative healing trajectories, cost-effectiveness, and real-world failure patterns is still insufficient. High-quality randomized trials and long-term follow-up will ultimately determine whether these innovations can truly surpass current standards.

Finally, long-term follow-up and comparative studies still need to be strengthened. Most of the current follow-up studies of all-inside reconstruction have focused on a 2- to 5-year period, suggesting that it has similar results to conventional techniques. However, ligament reconstruction long-term (>10 years) outcomes need to be supported by more data from high-quality randomized controlled trials and Meta-analyses. In particular, long-term effects such as arthritis prevention need to be studied in depth. To clarify the long-term safety and advantages of all-inside fixation devices, we propose initiating a r-RCT anchored in national ACL registries such as the MOON cohort or the Danish ACL Registry. A ≥10-year longitudinal backbone would first be established, after which patients would be randomly allocated to receive either an all-inside construct or a traditional full-tunnel (or alternative fixation) technique in a head-to-head comparison. This design minimizes loss to follow-up while maximising external validity. Sample size can be calculated using the IKDC minimal clinically important difference (MCID ≈ 6 points; SD ≈ 12 points): with *α* = 0.05 and *β* = 0.20, roughly 130 participants per arm are required. Follow-up assessments are planned at 2, 5, and 10 years—an approach informed by MOON 10-year data showing that tunnel widening and early osteoarthritic changes often emerge only after the 5-year mark. Core outcome domains: (1): Patient-reported outcomes: IKDC-2000, KOOS-JR, and ACL-RSI, capturing both functional status and psychological readiness to return to sport. (2): Objective stability: side-to-side KT-1000 difference ≤3 mm and instrumented pivot-shift with an acceleration threshold <0.5 m s^−2^. Both show moderate correlations with subjective function. (3): Imaging-based healing: quantitative MRI (T2/T1*ρ* or PDFF) of the graft–bone interface combined with 3-D CT measurements of tunnel volume. (4): Structural/failure events: graft revision, cyclops lesions, contralateral ACL rupture, and Kellgren–Lawrence grading, all of which determine long-term joint prognosis. (5): Health-economic endpoints: QALY-based cost–utility analysis and time to return to work or sport, essential for judging the true value of higher-cost devices. Current literature lacks standardized instrumented pivot-shift metrics, MRI-based quantitative healing thresholds, and real-world cost–effectiveness data; this proposed trial would provide high-quality evidence to fill these critical gaps.

### 9.2 Prospects

In the future, the all-inside reconstruction technique is expected to further improve and expand the indications with further research.

#### 9.2.1 Near-term outlook

In the foreseeable future, innovation will focus on fine-tuning current all-inside techniques. Precision socket-measurement tools (such as calibrated depth gauges and intra-operative ultrasound) will further reduce “bottom-out” events and early graft laxity. Biodegradable, self-locking high-strength button–loop devices have entered clinical trials, promising sustained suspensory strength without the need for later hardware removal. In addition, collagen-PLLA biosleeves combined with point-of-care PRP or BMAC injections are expected to shorten graft revascularization time (103). When paired with wearable-sensor home-rehabilitation programs, they may accelerate functional recovery while maintaining stability.

#### 9.2.2 Long-term outlook

Over a longer horizon, the convergence of materials science and digital surgery is poised to reshape all-inside reconstruction. Decellularized and recellularized tissue-engineered ligaments could replace autografts and allografts, providing “off-the-shelf” grafts with diameters of ≥8 mm. Magnesium-alloy resorbable buttons and PLGA adjustable loops equipped with load sensors will monitor *in vivo* tension in real time and degrade on demand (104). Robotic systems with haptic feedback, integrated with augmented-reality navigation, will position sockets with sub-millimetre accuracy in tight joint spaces, minimizing neurovascular risk. Deep-learning–based pre-operative planning will use imaging and demographic data to suggest the optimal graft strategy automatically, avoiding intra-operative surprises related to insufficient diameter (105). As these short-term refinements merge with long-term breakthroughs, all-inside reconstruction is likely to expand from the knee to the shoulder, ankle, hip, and other joints, continuing to drive the trend toward minimally invasive orthopaedic surgery.

## 10 Conclusion

As a great breakthrough achieved in orthopedics and other related medical fields in recent years, the all-inside reconstruction technique has been successfully applied to ACL/PCL reconstruction of the knee joint as well as soft-tissue reconstruction of the shoulder, hip, and other joints. A large number of related studies have confirmed that the total endoprosthesis technique is able to reduce surgical trauma and at the same time achieve the same mechanical stability and clinical efficacy as traditional surgery. The advantages include fewer grafts such as semitendinosus tendons, protection of the bone cortex, reduction of pain, faster rehabilitation, and facilitation of simultaneous multiple ligament reconstruction and revision surgery. In particular, the all-inside technique has been shown to be safe and effective in ACL reconstruction, with patients having better early pain and knee function than conventional procedures. All-inside reconstruction has also greatly facilitated the minimally invasive treatment of complex shoulder and hip injuries, and has improved the outcome of shoulder instability and acetabular labral injuries. Of course, there are still some shortcomings of the all-inside technique as well as areas that can be improved, such as optimizing the fixation device, preventing micromotion of the graft, and shortening the learning curve. In the future, more high-quality randomized controlled studies and long-term follow-up data are needed to further compare the similarities and differences between the all-inside technique and the traditional technique in terms of joint stability and prevention of joint degeneration. In conclusion, the all-inside reconstruction technique reflects the trend of “more minimally invasive without compromising the efficacy” of orthopedic surgery. With the accumulation of instruments and experience, it is expected that the all-inside technique will become one of the standard options for ligament reconstruction in all joints, providing patients with a safer and more effective treatment option.
